# Scavenged ^239^Pu, ^240^Pu, and ^241^Am from snowfalls in the atmosphere settling on Mt. Zugspitze in 2014, 2015 and 2016

**DOI:** 10.1038/s41598-017-12079-y

**Published:** 2017-09-19

**Authors:** Katharina Gückel, Taeko Shinonaga, Marcus Christl, Jochen Tschiersch

**Affiliations:** 1 Helmholtz Zentrum München, German Research Center for Environmental Health, Institute of Radiation Protection, Ingolstädter Landstr. 1, 85764 Neuherberg, Germany; 2 Laboratory of Ion Beam Physics, ETH Zurich, 8093 Zurich, TS Switzerland; 3 Present Address: Department of Radiation Chemistry, Institute of Radiation Emergency Medicine, Hirosaki University, Aomori, 036-8564 Japan

## Abstract

Concentrations of ^239^Pu, ^240^Pu, and ^241^Am, and atomic ratio of ^240^Pu/^239^Pu in freshly fallen snow on Mt. Zugspitze collected in 2014, 2015 and 2016 were determined by accelerator mass spectrometry (AMS). For the sub-femtogram (10^−15^ g) - level of Pu and Am analysis, a chemical separation procedure combined with AMS was improved and an excellent overall efficiency of about 10^−4^ was achieved. The concentration of ^239^Pu ranges from 75 ± 13 ag/kg to 2823 ± 84 ag/kg, of ^240^Pu from 20.6 ± 5.2 to 601 ± 21 ag/kg, and of ^241^Am was found in the range of 16.7 ± 5.0–218.8 ± 8.9 ag/kg. Atomic ratios of ^240^Pu/^239^Pu for most samples are comparable to the fallout in middle Europe. One exceptional sample shows a higher Pu concentration. High airborne dust concentration, wind directions, high Cs concentrations and the activity ratio of ^239+240^Pu/^137^Cs lead to the conclusion that the sample was influenced by Pu in Saharan dust transported to Mt. Zugspitze.

## Introduction

Since the first nuclear weapons test (Trinity test) performed in New Mexico, USA in 1945, plutonium (Pu) and americium (Am) have been released into the environment through various events. It is assumed that around 1.4 ∙ 10^16^ Bq of ^239+ 240^Pu have been released into the environment until now^1,2^. The sources are continued nuclear weapons tests (1.2 ∙ 10^16^ Bq)^1^, accidents at nuclear facilities (2.65 ∙ 10^15^ Bq)^2,3^, crashes of satellites (6.3 ∙ 10^14^ Bq ^238^Pu) and planes, and discharging from reprocessing factories (>5.8 ∙ 10^14^ Bq)^4^. ^241^Am (T_1/2_: 432.2 a), is the beta-decay product of ^241^Pu (T_1/2_: 14.325 a)^5^, and its concentration in the environment continues to increase. It was estimated that ^241^Am would reach its maximum activity in the middle of the 21^st^ century, supposing no further significant releases would be happened^6^. The major isotopes of Pu and Am are alpha emitters with long half-lives (^239^Pu: 24,110 a, ^240^Pu: 6563 a), which lead to long residence times in the environment^7,8^. Pu and Am are not considered essential elements for the human body and internal contamination thereof may result in radiological and chemical hazard^9^. After incorporation of Pu into the human body, it is rapidly deposited in bones and liver^9^. The biological half-life is 50 years in bones and 20 years in liver^10^.

For the forecast of long-term radiological consequences of an accidental release of toxic actinides into the environment, it is important to understand the behaviour of those elements in the environment. Actinides contained in wet deposition are one of the sources for incorporation in the hydrosphere. Although some studies relating to actinides in rains accumulated on level lands have been reported^11,12^, limited data of the Pu and Am wet deposited in the alpine area exists and details on the transport of the actinides from alpine area into the hydrosphere are still not entirely understood^11,13–15^. In a study of an ice core in the alpine area, contamination of ^239^Pu in the Swiss/Italian Alps was analysed without chemical separation using ICP-MS^13^. It was found that the ^239^Pu profile had three peaks caused by nuclear weapon tests after 1950s and therewith high Pu concentrations. Transport and behaviour of Pu fallout in surface waters were also studied^15–17^. They derived that the main source of Pu in surface waters in the northern hemisphere is the global fallout originating from the nuclear weapons test in the 1950s and 1960s.

In this study, we analysed the quantity of Pu isotopes and ^241^Am and the atomic ratio of ^240^Pu/^239^Pu in snowfalls on Mt. Zugspitze to obtain the data of those nuclides scavenged from snowfalls in the alpine area. For the sub-femtogram (10^−15^ g) - level of Pu and Am analysis, a chemical separation procedure using UETVA and TRU extraction chromatography resins was improved and its reliability was tested.

For the measurement of isolated Pu and Am the compact accelerator mass spectrometer, AMS TANDY at the Laboratory of Ion Beam Physics, ETH Zurich, Switzerland was used^18^. The ETH Zurich 0.6 MV Tandy AMS system has been optimized for the determination of ultra-trace levels of actinides^19^, it combines very high sensitivity and substantial background suppression providing detection limits at the sub-femtogram level (i.e. <10^−15^ g)^20^. With a critical level of 40 × 10^−18^ g for ^241^Am (Table 1) compact AMS provides an about one order of magnitude higher sensitivity than conventionally applied counting techniques even for the comparably short lived radionuclide ^241^Am (T_½_ = 432.2 a)^21^. The total number of ^239^Pu atoms in an about 100 kg snow sample was typically at the order of 10^7^. From these samples typically >1000 counts were registered in the detector on mass 239. This implies an excellent overall efficiency (including all steps of chemical preparation, incomplete sample consumption during AMS measurement, and other causes) of about 10^−4^. Under these conditions Pu and Am concentrations at the sub-femtogram level were determined.

**Table 1:** Error weighted averages of blank values, u_c_: combined uncertainty; at: number of atoms, n: number of samples, L_C_: critical limit for detection (L_C_ = 1.645 × 2u_c_), fg: 10^−15^g.

Blank Atomic ratio	Error weighted average (at/at)	u_c_ (at/at)	n	Snow samples (min.–max.: at/at)	Lc (fg)
^239^Pu/^242^Pu	0.00642	0.00033	6	0.0106–0.2779	^239^Pu: 0.75
^240^Pu/^242^Pu	0.00104	0.00022	6	0.0098–0.0586	^240^Pu: 0.49
^241^Am/^243^Am	0.000123	0.000018	5	0.0012–0.0194	^241^Am: 0.04

This study provides for the first time results of the amount of Pu and Am scavenged from snowfalls settled on Mt. Zugspitze during the snow season of 2014–2015 and 2015–2016. The obtained results might contribute to understanding the distribution and the behaviour of those actinides in the atmosphere-snow-hydrosphere system in alpine areas.

## Sampling and Experimental Method

### Sampling Area

**Figure 1:**
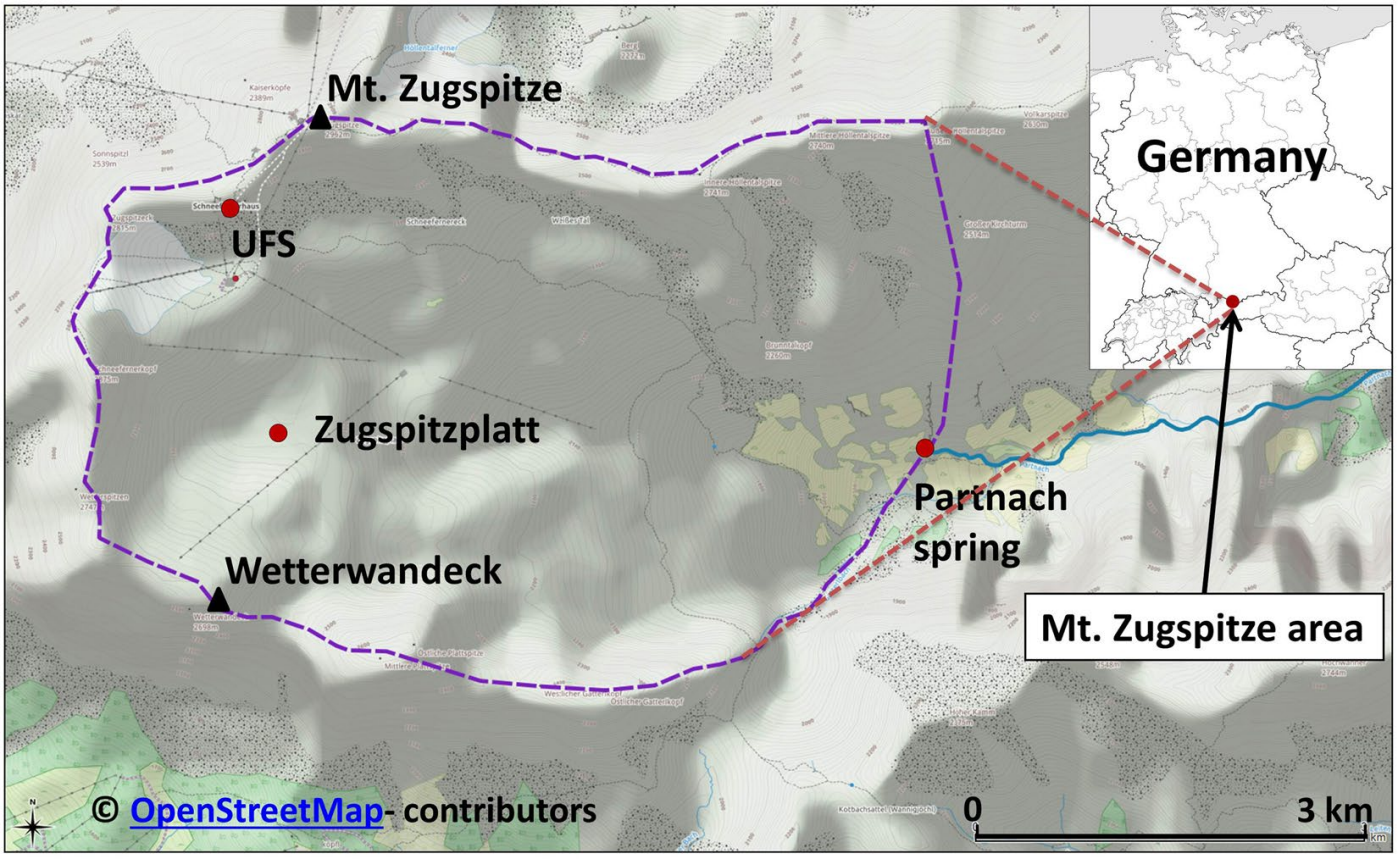
Catchment of Mt. Zugspitze (purple line), Partnach spring and sampling areas UFS and Zugspitzplatt^22^. Map data ^©^OpenStreetMap contributors (www.openstreetmap.org/copyright).

Sampling of undisturbed freshly fallen snow (60–145 kg snow) was carried out at the Environmental Research Station Schneefernerhaus (UFS) on Mt. Zugspitze, 2650 m above sea level (a.s.l.). The UFS is located at the south slope of Mt. Zugspitze, which is part of the Wetterstein Mountains in the South of Germany. Additionally, naturally accumulated snow was sampled on the Zugspitzplatt near the Wetterwandeck, 2420 m a.s.l. In Fig. 1 the location of Mt. Zugspitze, the catchment with the Partnach spring, and the sampling areas UFS and Zugspitzplatt are illustrated.

### Snow Sampling

Undisturbed freshly fallen snow (SZXX, Table 2) in the snow season of 2014–15 (Oct.–April) and 2015–16 (Nov.–May) were collected in 40 L high-density polyethylene (HDPE) wide-necked barrels, whose inner surface was acidified with 65% HNO_3_. Sampling point was a terrace of the UFS, which was cleaned of snow after every snowfall to ensure collecting only freshly fallen snow timely. Additionally, naturally accumulated snow (JZP 14/15, JZP 15/16) was sampled as a bulk sample down to near the ground at the Zugspitzplatt. The sampling dates and collected quantities of snow are given in Table 2. The densities were determined by collecting undisturbed snow in a pipe with a known volume and weighing of this snow.

**Table 2:** Sampling date, amount and density of collected snow.

Nr.	Sample name	Sample date	Mass [kg]	Density [kg/m³]
1	SZ 23.10.14	Oct. 23. 2014	69.7	116
2	SZ 12.01.15	Jan. 12. 2015	109.3	181
3	SZ 29.01.15	Jan. 29. 2015	72.8	135
4	SZ 27.11.15	Nov. 27. 2015	60.6	22.5
5	SZ 24.02.16	Feb. 24. 2016	104.2	139
6	SZ 26.02.16	Feb. 26. 2016	101.0	225
7	SZ 01.03.16	March 01. 2016	73.2	147
8	SZ 24.03.16	March 24. 2016	66.2	120
9	SZ 14.04.16	April 14. 2016	85.9	159
10	^a^JZP 14/15	May 13. 2015	143.6	522
11	^b^JZP 15/16	May 03. 2016	103.0	453

SZ: fresh snowfall, ^a^JZP: snow naturally accumulated in the snow season of 2014–2015 and ^b^2015–2016.

### Sample Preparation

The complete snowmelt (about 100 L, see Table 2) was acidified with concentrated HNO_3_ (~pH 1, ~800 mL) to prevent actinide sorption on the barrel surface and pre-concentrated to 100 mL in a closed large capacity evaporator (Heidolph Laborota 20 control) at 95 °C. After adding around 1 pg of each spike the samples were evaporated to dryness to remove HNO_3_ and afterwards dissolved in iron sulfamate/4 M HNO_3._ The samples were filtered before the chemical separation. Process blanks (100 L spiked distilled water, ~800 mL HNO_3_) were treated in the same way.

### Analysis of Certified Reference Material, IAEA-443 (Irish Sea Water)

The reliability of the analytical results of Pu and Am obtained with AMS was examined using the certified reference material IAEA-443 (Irish Sea water). For the determination of the Pu isotopes and ^241^Am 500 mL homogenised reference material were acidified with HNO_3_ (pH 1) and spiked with about 1 pg of each tracer (^242^Pu, ^243^Am). 2 mg iron carrier was added for the co-precipitation by iron hydroxide. Iron was precipitated by adding ammonium hydroxide until pH 9 was reached. The solution was subsequently decanted and the precipitate centrifuged and washed thoroughly with distilled water. Before the chemical separation, the precipitate was dissolved in 10 mL iron sulfamate/4 M HNO_3_.

### Chemical Separation

For the low concentration analysis of Pu and Am chemical isolation from matrix and interferences is necessary. In literature, numerous methods were reported and the chemical separation with extraction chromatography resins is widely used for environmental samples^23–29^.

One of the advantages of ^239^Pu measurements with compact AMS at ETH Zurich is the good abundance sensitivity (at the order of 10^−12^) that minimizes potential interferences from the neighbouring mass ^238^U. To further minimize such influence, U was extracted from Am and Pu at the first stage of their chemical separation. For this propose the extraction chromatography resin UTEVA^®^ (Triskem International) is commonly used and the methods were well studied^28–32^. Pu and Am in the 3+ oxidation state are not retained on the resin UTEVA^®^ and are separated using the extraction chromatography resin TRU^®^ (Triskem International).

In literature, various procedures for the separation of actinides with TRU^®^ resin are presented^26,27,29,31,33,34^. Although 5 to 10 mL of 4 M HNO_3_ is commonly used for equilibration of the resin and rinsing/washing after the sample loading, various separation procedures of Pu and Am were examined to obtain better chemical yield using radioactive tracers (^242^Pu IRMM-085 and ^243^Am NIST 4332E) and liquid scintillation counting. Eluting with 9 M and 4 M HCL for Am and with 0.1 M Ammoniumbioxalate for Pu result in the highest chemical yield. The chemical yield was 88% for Pu and 77% for Am in average (number of analysis: 5, scheme see Supplementary information).

The chemical separation of Pu and Am in the snow samples and process blanks was performed adding about 1 pg of spike ^242^Pu (IRMM-085) and ^243^Am (NIST 4332E) as internal standard. After the elution, the actinides were co-precipitated with ironhydroxide and transferred into the oxide form in a muffle furnace at 600 °C for 4 h. Then, the sample material consisting of Actinide oxide and Fe_2_O_3_ is mixed with Nb powder and pressed into Ti sample holders (cathodes) for the AMS measurements.

### AMS at ETH Zurich

The AMS measurements were performed with the compact (0.6 MV) AMS system TANDY at ETH Zurich. The AMS set-up for actinide measurements at ETH Zurich has been described in detail elsewhere^18,35^. Negatively charged actinides oxide ions are extracted from the Cs-sputtering and injected into the accelerator running at a terminal voltage of about 300 kV. At the terminal, helium is used as a stripper gas to break up the injected molecules and to generate positively charged actinides ions. Generally, the 3^+^ charge state is selected on the high energy side for all actinides because it provides highest stripping efficiencies of about 35%. In the last step, ion identification is made with a dedicated low noise gas ionization detector. In our study ^241^Am was analysed relative to the ^243^Am tracer and Pu isotopes relative to the ^242^Pu tracer. Typical counting rates for about 1 pg of both tracers were 150 cps. All Pu ratios are normalized to the ETH Zurich in house standard “CNA” and ^241^Am/^243^Am ratios are normalized to an in house prepared standard containing known amounts of ^241^Am and ^243^Am^18^. All measured ratios are corrected with error weighted averages of blank values (Table 1). The Am fraction was additionally analysed on mass 242 to detect a potential carry-over of Pu. The average ^242^X/^243^Am ratio of all snow samples presented in this study was 0.0024, indicating that the isobar problem due to ^241^Pu for the measurement of ^241^Am is negligible for the method used in this study.

## Results and Discussion

### Analytical Results of Certified Reference Material, IAEA-443 (Irish Sea Water)

The results of the analysis and the reference values are given in Table 3. The combined uncertainties of the concentrations and atomic ratio were calculated according to “Evaluation of measurement data - Guide to the expression of uncertainty in measurement” (GUM)^36^ and expressed as expanded uncertainties (U) with coverage factor 2 (k = 2).

**Table 3:** Analytical results of Pu isotopes and ^241^Am in IAEA-443 (Irish Sea water).

Sample name	^239^Pu ± U (fg/kg)	^240^Pu ± U (fg/kg)	^240^Pu/^239^Pu ± U	^241^Am ± U (fg/kg)	Snow samples (see Table 1)
IAEA443-1	3674 ± 74	915 ± 18	0.2480 ± 0.0070	187 ± 19	1–3, 10
^a^Predicted value	3564 ± 397	833 ± 91	0.233 ± 0.072	167.1 ± 7.9	
IAEA443-2	3732 ± 94	900 ± 23	0.2400 ± 0.0089	169.1 ± 5.4	4–9, 11
^b^Predicted value	3564 ± 397	833 ± 91	0.233 ± 0.072	168.0 ± 8.0	
IAEA443-3	3749 ± 95	916 ± 23	0.2434 ± 0.087	n.a.	
^c^Predicted value	3564 ± 391	833 ± 83	0.234 ± 0.069	n.a.	

IAEA443-1,2,3: this study; ^a^Predicted value: as of July 30. 2015; ^b^Predicted value: as of June 10. 2016; ^c^Predicted value, as of Nov. 24. 2016; U, expanded uncertainty (k = 2); n.a., not analysed; snow sample number (see Table 1) analysed together with indicated reference material.

A Chi-Squared test performed for each sample pair (measurement vs. reference value) shows that none of the single measurements (^239^Pu, ^240^Pu, and ^241^Am, and ^240^Pu/^239^Pu ratio) is significantly different from the reference value. A Chi-Sq. above 3.84 (2 samples, 1 degree of freedom, 95% confidential) would indicate that two sample are distinguishable from each other. Chi-Sq. values between 0.01 and 2.46 were calculated for the data.

### Concentration of ^239^Pu and ^240^Pu in Snows on Mt. Zugspitze

**Figure 2:**
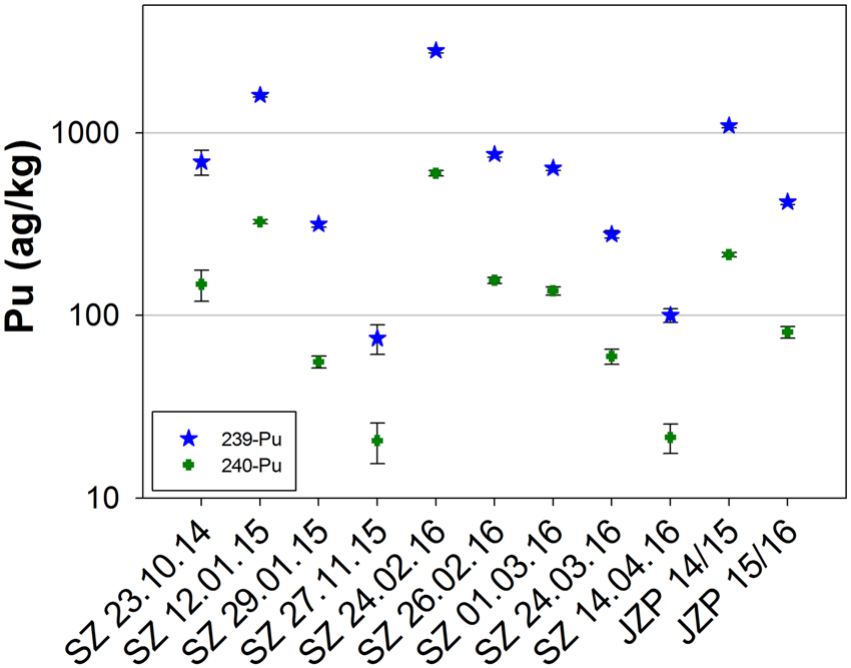
Concentrations of ^239^Pu (blue star) and ^240^Pu (green cross) in the snow samples. The error bars indicate expanded uncertainty (k = 2). SZ: freshly fallen snow Zugspitze, JZP: naturally accumulated snow.

In Table 4 and Fig. 2 the concentrations of ^239^Pu and ^240^Pu in the snow sampled at Mt. Zugspitze are given.

**Table 4:** Analytical results of ^239^Pu,^239^Pu and ^241^Am concentration and atomic ratio of ^239^Pu/^240^Pu in snow samples.

Sample name	^239^Pu (ag/kg)	U (k = 2) (ag/kg)	^240^Pu (ag/kg)	U (k = 2) (ag/kg)	^240^Pu/^239^Pu	U (k = 2)	^241^Am(ag/kg)	U (k = 2) (ag/kg)
SZ 23.10.14	693	108	148	29	0.213	0.053	16.7	5.0
SZ 12.01.15	1611	42	325.5	8.7	0.2013	0.0075	137.4	7.9
SZ 29.01.15	316	11	55.9	4.1	0.176	0.015	218.8	8.9
SZ 27.11.15	75	13	20.6	5.2	0.274	0.085	89.6	7.4
SZ 24.02.16	2823	84	601	21	0.2119	0.0097	156	19
SZ 26.02.16	763	26	155.5	6.0	0.203	0.011	40.5	2.5
SZ 01.03.16	640	19	136.1	7.1	0.21	0.013	63.3	3.6
SZ 24.03.16	278	14	59.9	5.8	0.214	0.023	43.7	3.5
SZ 14.04.16	100.0	8.7	21.5	3.9	0.214	0.043	17.3	1.9
JSZ 14/15	1094	29	215.0	5.8	0.1956	0.0074	126.1	5.1
JZP 15/16	418	15	80.9	6.0	0.193	0.016	207.2	7.5

U: expanded uncertainty.

Analytical results for Pu in the process blank showed below detection limit. The concentrations of ^239^Pu (blue circles) in the freshly fallen snow range from 75 ± 14 ag/kg to 2823 ± 84 ag/kg. For the naturally accumulated snow during the snow season in 2014–2015 and 2015–2016, the concentrations were obtained as 1094 ± 29 and 418 ± 15 ag/kg, respectively. For ^240^Pu (green cross) concentrations values between 20.6 ± 5.2 to 601 ± 21 ag/kg were obtained in the freshly fallen snow and for the naturally accumulated snow of the snow season in 2014–2015 and 2015–2016, 215.0 ± 5.8 and 80.9 ± 6.0 ag/kg, respectively, were found. Differences in the concentrations in the naturally accumulated snow samples may occur because the snow occasionally evaporated, sublimated or melted. Pu may be incorporated in the snowmelt and removed from the snow bed due to melting processes resulting in a concentration decrease. In contrast, evaporation and sublimation of snow leads to an enrichment of Pu in the remaining snow. However such events cannot be clarified in this study.

The ^239+240^Pu activity concentration scavenged from snow in the atmosphere of Mt. Zugspitze determined in this study ranges from 0.35–11.54 µBq/kg and this corresponds to the activities found in other matrices affected by global fallout, like spring water, rain, and lake water. The input of radioactive fallout on spring water from the Venoge karst system was analysed and a ^239+240^Pu activity concentration range of 4.3–22.8 µBq/L was found^37^. In rain collected in Monaco in the years 1999 to 2001^239+240^Pu activities between 1.5 and 430 µBq/L were found^38^. In surface water of Lake Päijanne in Finland, which received a significant deposition from Chernobyl fallout, a ^239+240^Pu activity concentration of 4.9 µBq/L was found^39^.

The highest activity concentration of 11.54 µBq/kg obtained in the fresh snow sample SZ 24.02.16 was considered as an influence of Sahara dust according to wind direction and air particle level. The detailed discussion on this is described in section **Influence of Particles Transported by Wind**. Without this input the activity concentration of ^239+240^Pu ranges from 0.35 ± 0.11 to 6.44 ± 0.24 µBq/L. The results indicate that ^239+240^Pu concentrations scavenged from snow in the atmosphere on Mt. Zugspitze are at the same level as those in other compartments affected by global fallout.

### Atomic Ratio of ^240^Pu/^239^Pu

**Figure 3:**
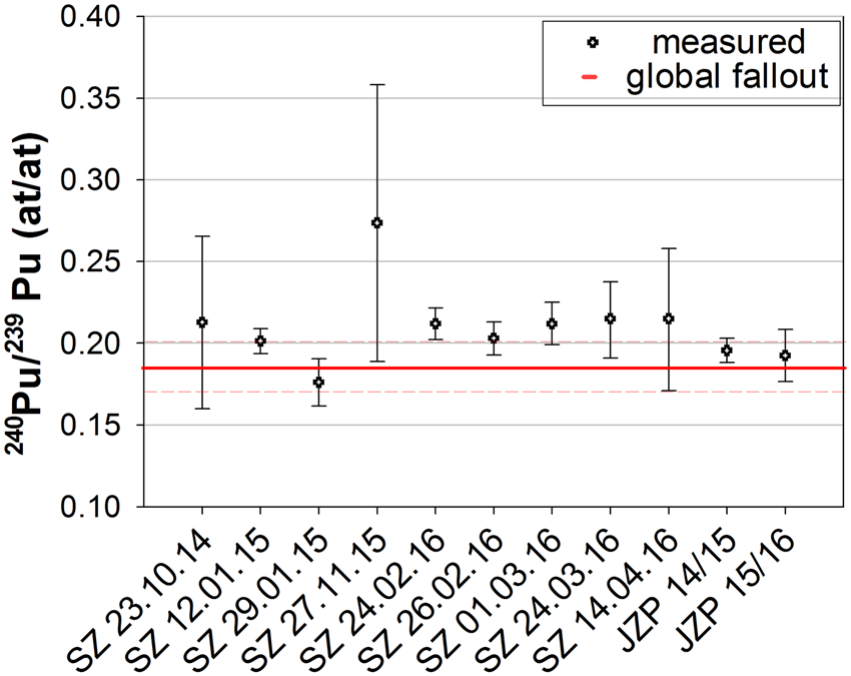
Atomic ratio ^240^Pu/^239^Pu for the snow samples and value of global fall in middle Europe^47^ (red line). The error bars indicate expanded uncertainty (k = 2). SZ: freshly fallen snow Zugspitze, JZP: naturally accumulated snow.

To understand the sources of Pu in snow the atomic ratio ^240^Pu/^239^Pu was calculated. Previous studies reported the ratios of ^240^Pu/^239^Pu in particles as 0.33–0.56^40–43^ from Chernobyl, as 0.18–0.19 from nuclear weapons test fallout^4^, and as 0.22–0.25^44–46^ and 0.34^47^ in effluents from the reprocessing plant in Sellafield and La Hague, respectively. The atomic ratios ^240^Pu/^239^Pu of the snow samples are shown in Fig. 3. The obtained ratios are in the range of the fallout in middle Europe (^240^Pu/^239^Pu = 0.17–0.19^48^) taking the uncertainties into account.

### Influence of Particles Transported by Wind

**Figure 4:**
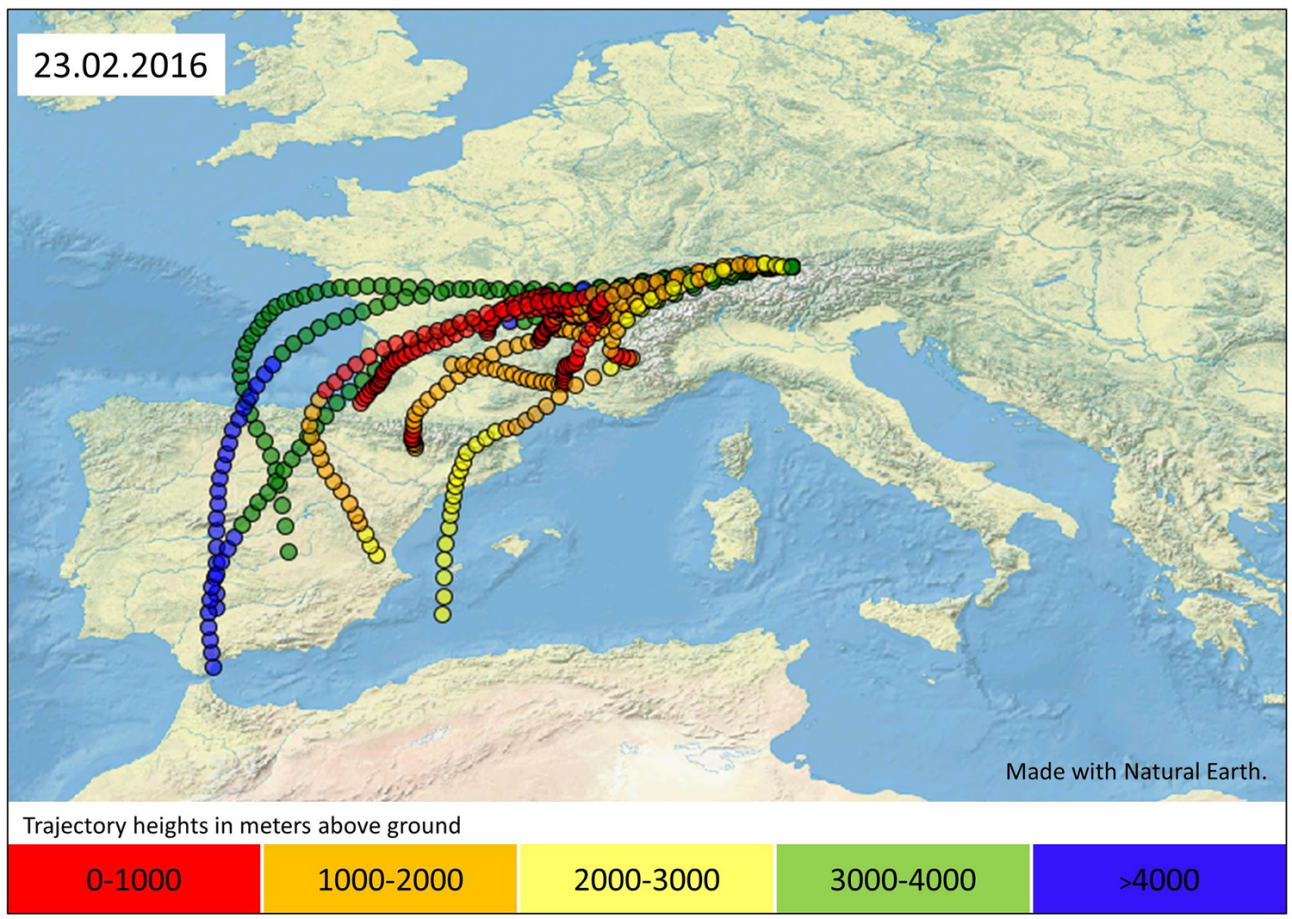
Backward trajectories arriving at Umweltforschungsstation Schneefernerhaus on the 23.02.2016 between 0 and 10 am, computed using the FLEXTRA code. Meteorological driver is the Global Forecast Model. Made with Natural Earth^62^.

For the sample SZ 24.02.2016, which has the highest Pu concentration, an influence of Saharan dust is assumed. The sample contains 38 µg/kg dust particles washed out by snow compared to <1 µg/kg in the other samples. A large value of PM10 concentration of 40 µg/m³ (atmospheric particulate matter <10 µm) (average <5 µg/m³)^49^ was recorded at the Umweltforschungsstation Schneefernerhaus (UFS) on February 23^rd^ 2016 caused by an airborne transport of material originating from the south, as was indicated by the modelled backward trajectories of the 23.02.2016, 0–10 am (Fig. 4). Backward trajectories for the other sampling dates were also modelled to check for Saharan dust influence. The trajectories do not originate from the south and therefore no Saharan dust influence was indicated.

A recent study relating to the Fukushima Daiichi Nuclear Power Plant indicates that Pu was transported to 120 km distance by wind, representing the isotopic ratio of Pu as evidence^50^. Other studies determined the Saharan region as the world´s largest source for dust particles transported by wind^51–53^ with 80 to 120 ∙ 10^6^ tons/a of dust particles transported towards Europe^54^. It was reported that Saharan dust, spread over Europe, increased the radionuclide concentration in air significantly^52,55^. Radioactive particles of the global fallout remained in the upper sediment layer in the arid Saharan region for a very long time and can be remobilised by wind and transported over long distances^13^. Wagenbach and Geis^56^ demonstrated that two-thirds of the deposited dust in the Southern Alps originate from the Saharan desert. In the early 1960s France conducted several nuclear bomb tests in the south of Algeria. The International Atomic Energy Agency (IAEA) analysed soil and sand samples from the test sides and found ^239+240^Pu activity concentrations up to 1.2∙10^6^ Bq/kg^57,58^. Although it was shown that the Pu concentration in particles is higher than in the global fallout, the atomic ratios of ^240^Pu/^239^Pu measured in Saharan dust indicate contamination from global fallout^38,52,53,55^.

For further clarification, the activity concentration of ^137^Cs in the red coloured filter residue (>0.1 µm) of sample SZ 24.02.16 was analysed by gamma spectrometry with a high-purity Germanium detector. A quantity of 0.261 ± 0.019 mBq/kg ^137^Cs was found in the sample and an activity ratio ^239+240^Pu/^137^Cs of 0.044 ± 0.004 was obtained. This value is higher than the global fallout measured in Bavaria and the European alpine region of 0.018^59,60^_._ According to literature^38,61^, the value of ^239+240^Pu/^137^Cs found in this study agrees with that measured in rain and surface water samples containing Saharan dust (0.024–0.04) collected in Monaco and on the Mediterranean Sea. This implies that the snow sampled on 24.02.16 contained Saharan dust, transported to the European alpine area. This indicates that an additional input of actinides into the snow-hydrosphere system may have occurred in addition to the global fallout.

### Concentration of ^241^Am in Snow

**Figure 5:**
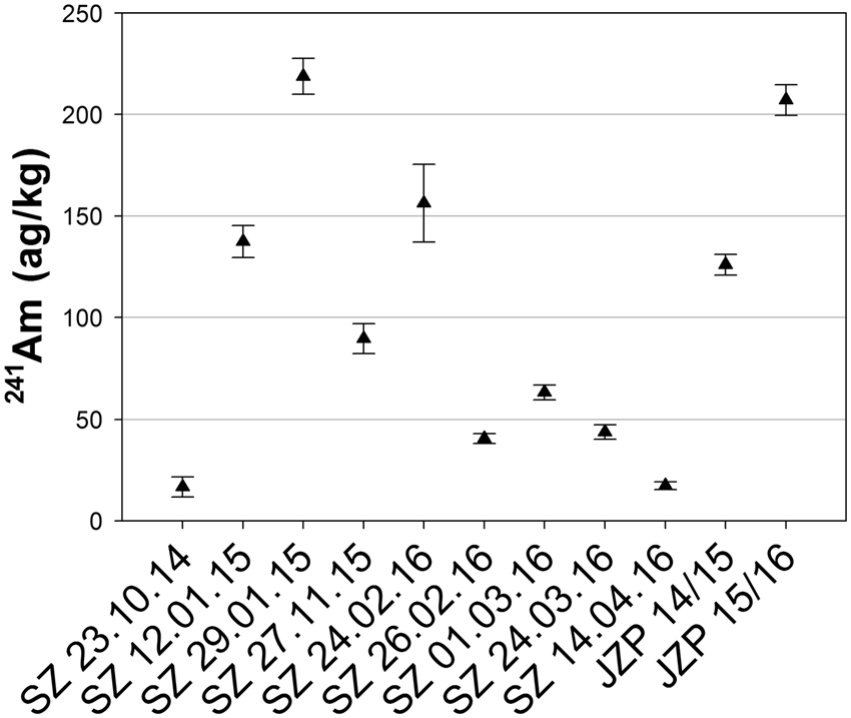
^241^Am concentrations in the snow samples. The error bars indicate expanded uncertainty (k = 2). SZ: freshly fallen snow Zugspitze, JZP: naturally accumulated snow.

Analytical results for Am in the process blank showed below detection limit. The samples of freshly fallen snow contain 16.7 ± 5.0–218.8 ± 8.9 ag/kg ^241^Am. For the naturally accumulated snow of the snow season in 2014–2015 and 2015–2016, 126.1 ± 5.1 and 207.2 ± 7.5 ag/kg ^241^Am, respectively, were obtained (Table 4 and Fig. 5).

To the best of our knowledge, these are the first data of Am concentrations in snowfalls. The concentrations vary throughout the years and no clear pattern is visible. Slightly higher concentration in the freshly fallen snow in the period from November 2015 to April 2016 in the sample SZ 24.02.16, which also has a high PM10 particle concentration^48^, may be caused by input of particles with high Am concentration in Saharan dusts^38,61,63^. Although France conducted several nuclear bomb tests in the south of Algeria in the early 1960s and ^241^Am activity concentrations up to 2.4∙10^4^ Bq/kg^57,58^ are found in soil and sand samples from the test sides, the activity ratios ^241^Am/^239+240^Pu and atomic ratios ^240^Pu/^239^Pu of samples containing Saharan dust are within the variation of global fallout^38,52,61^. Furthermore, the Pu and Am concentrations are increased in these samples. Matching results are found for sample SZ 24.02.16. The Pu and Am concentrations are increased by input of Saharan dust and an ^241^Am/^239+240^Pu activity ratio (1.72 ± 0.21) in the range of global fallout is found.

The ^241^Am activity concentration in snow on Mt. Zugspitze determined in this study ranges from 2.12 ± 0.63 to 27.8 ± 1.1 µBq/kg. The input of radioactive fallout into soil water and spring water from the Venoge karst system leads to ^241^Am activity concentrations of 1.6–2.3 µBq/L in soil water and of 1.2–2.9 µBq/L in spring water^37^. In surface water of Lake Päijanne in Finland, which received a significant deposition from Chernobyl fallout, an average Am activity concentration of 4.1 µBq/L was found^39^. ^241^Am activity concentrations between 1.3 and 150 µBq/L in rain collected in Monaco in the years 1999 to 2001 were found^38^. The results of snow obtained in this study are within those ranges.

### Summary

For the first time concentrations of ^239^Pu, ^240^Pu, and ^241^Am and the atomic ratio of ^240^Pu/^239^Pu in freshly fallen snow and naturally accumulated snow on Mt. Zugspitze collected in 2014, 2015 and 2016 were determined by accelerator mass spectrometry (AMS). For the sub-femtogram (10^−15^ g) - level of Pu and Am analysis, a chemical separation procedure combined with AMS was improved and an excellent overall efficiency of about 10^−4^ was achieved. The concentration of ^239^Pu in freshly fallen snow and naturally accumulated snow ranges from 75 ± 13 ag/kg to 2823 ± 84 ag/kg, of ^240^Pu from 20.6 ± 5.2 to 601 ± 21 ag/kg and ^241^Am was found in the range of 16.7 ± 5.0–218.8 ± 8.9 ag/kg. Atomic ratios of ^240^Pu/^239^Pu for most samples are comparable to the global fallout in middle Europe. For one exceptional sample, which shows a higher Pu concentration, an influence of Pu in dusts transported from the Sahara Desert was considered. Dust loads, wind directions, high Cs concentrations and the ^239+240^Pu/^137^Cs strengthen this assumption. The results give a first impression about the amounts of Pu isotopes and ^241^Am deposited on and with snow at Mt. Zugspitze.

## Electronic supplementary material

Scheme of the chemical separation
