# Supplementary material for: Scavenged 239Pu, 240Pu, and 241Am from snowfalls in the atmosphere settling on Mt. Zugspitze in 2014, 2015 and 2016
Source: Sci Rep. 2017 Sep 19;7:11848. doi: 10.1038/s41598-017-12079-y (PMC5605513; doi:10.1038/s41598-017-12079-y)
Supplement: Supplementary file 1 — Scheme of the chemical separation [file 41598_2017_12079_MOESM1_ESM.pdf]

Scavenged ^{239}Pu, ^{240}Pu, and ^{241}Am from snowfalls in the atmosphere settling on Mt. Zugspitze in 2014, 2015 and 2016

Katharina Gückel[1], Taeko Shinonaga[1], Marcus Christl[2] & Jochen Tschiersch[1]

[1]Helmholtz Zentrum München, German Research Center for Environmental Health, Institute of Radiation Protection, Ingolstädter Landstr. 1, 85764 Neuherberg, Germany. [2]Laboratory of Ion Beam Physics, ETH Zurich, 8093 Zurich, Switzerland. T.S[1], present address: Department of Radiation Chemistry, Institute of Radiation Emergency Medicine, Hirosaki University, Aomori 036-8564, Japan. Correspondence and requests for materials should be addressed to K.G. (email: katharina.gueckel@helmholtz-muenchen.de)

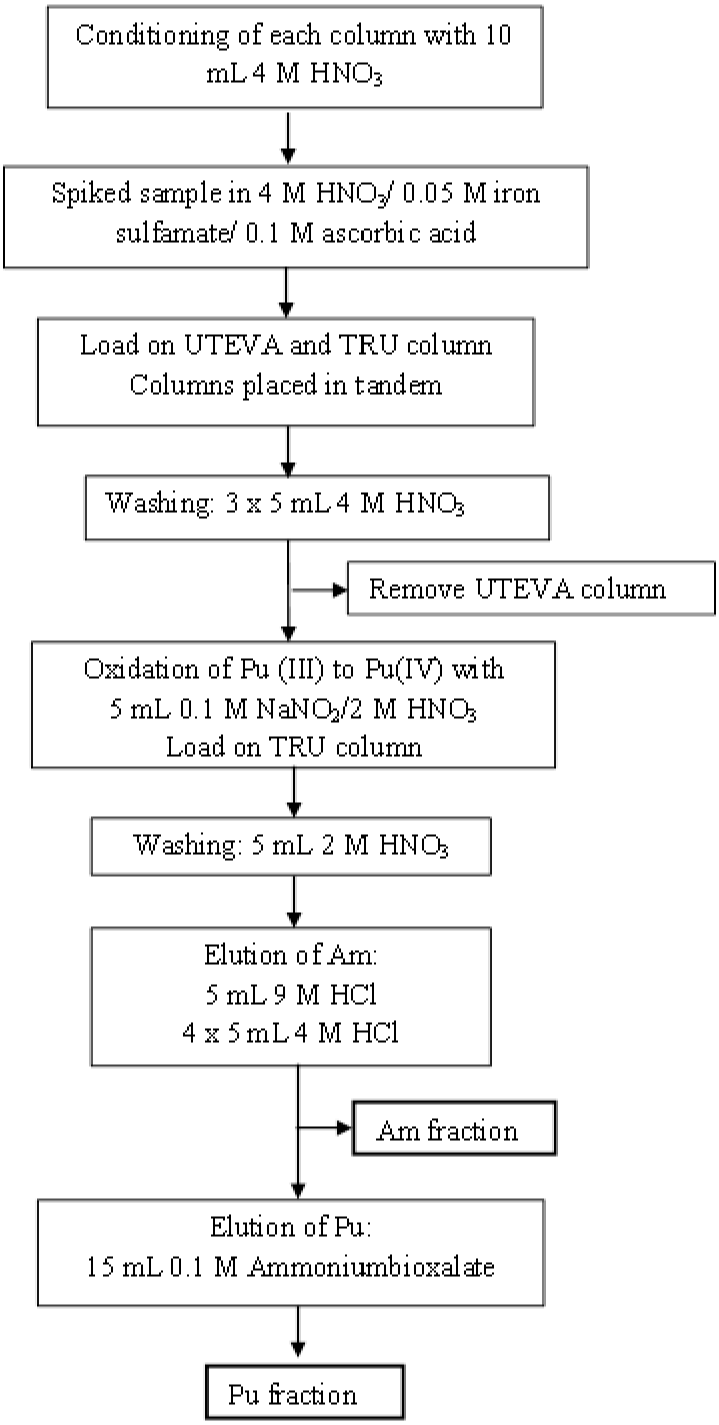

Figure Sub1. Scheme of the chemical separation for Pu and Am with UTEVA®- and TRU® resin
